# Assessing the Empirical Linkage Among Access to Finance, Firm Quality, and Firm Performance: New Insight From Bangladeshi SMEs’

**DOI:** 10.3389/fpsyg.2022.865733

**Published:** 2022-04-27

**Authors:** Mohammad Abir Shahid Chowdhury, Shuai Chuanmin, Marcela Sokolová, Ahsan Akbar, Zahid Ali, Hussain Ali, Md. Zahid Alam

**Affiliations:** ^1^School of Economics and Management, China University of Geosciences, Wuhan, China; ^2^Department of Management, Faculty of Informatics and Management, University of Hradec Králové, Hradec Králové, Czechia; ^3^International Business School, Guangzhou City University of Technology, Guangzhou, China; ^4^Department of Commerce and Management, University of Malakand, Chakdara, Pakistan

**Keywords:** access to external finance, firm performance, small- and medium-sized enterprises (SMEs), propensity score matching techniques, sustainable firm growth

## Abstract

Access to finance plays a central pillar on the sustainable firm growth of developing and developed nations. This study depicts the linkage between access to external finance, firm quality, and firms’ performance as measured by labor productivity for sustainable small- and medium-sized enterprises (SMEs’) development by employing the ordinary least squares (OLS) regression and propensity score matching (PSM) techniques to alleviate the selection bias and endogeneity issue on Word bank enterprise survey (WBES) cross-sectional firm-level data of 3,196 Bangladeshi SMEs for 2007–2013 period. Empirical evidence linking access to external finance and labor productivity has been positive and significant. However, our finding explores a negative but significant relationship between exports and SME labor productivity. A further look into the results also exhibits no statistical significance in the interaction effect between firm quality and access to finance on labor productivity. Moreover, the study anticipates novel empirical support that, the disintegration effect of export sales between direct and indirect exports with labor productivity for credit-accessed firms, is also found statistically insignificant. Then, several policies are drawn from the results to gain international competitiveness, and to ensure more external finance channels for enhancing SMEs’ performance and sustainable firm growth.

## Introduction

It is well established that SMEs are the central participants in developing economies that urge researchers to invent and apply several policies to promote small- and medium-sized enterprises’ development and enlargement (Rupasingha and Wang, 2017). However, the recent world economic situation has pointed out that SMEs’ access to finance is a vital pillar to identify SMEs’ sustainable firm performance, in particular labor productivity (Dollar et al., 2005; Ayyagari et al., 2008).

A large body of the literature acknowledged that access to finance accelerates enterprise-level investments and expedites tangible and labor capital aggregation, as well as the modernization and adoption of advanced technologies, affirmatively influencing enterprise productivity and growing firms’ productivity (Beck et al., 2000; Redmond and Van Zandweghe, 2016; Giang et al., 2019). It is conceded as a key pillar in stimulating firms’ growth and sustainability through investing in both ongoing and novel projects. In particular, firms will finance in a venture where the forecasted profit surpasses the expenses. Nonetheless, firms can make an optimum investment when they do not encounter liquidity constraints linked to their productivity (Hallward-Driemeier et al., 2006; Hoewer et al., 2012). On the contrary, several literatures highlighted that lack of access to external finance may lead to lower productivity and create obstacles in the larger participation of firms. The viable reason for the insufficient possibility of access to finance is due to the absence of information symmetries between lenders and borrowers, even though confidential data or information is available to the borrowers regarding firm quality. Credit rationing in the market may happen due to this unavailability of access to finance (Craig et al., 2007). In this scenario, Smaller and newly established firms may apply lending instruments such as fixed-asset collateral agreement and externally amended financial statements to signal firm quality when financial institutions have mere information about firms’ operating activities (Mwangi, 2008). In this context, small and medium-sized firms may depend on exports on the unavailability of the lending instrument to exhibit firm quality since this may signal better quality to invest.

Therefore, in this regard, whether firms have good quality to finance is complicated to analyze the relationship between productivity and credit access as the international entry of SME, *via* international market sales information, may show competitiveness and efficiency of SME. A firm’s exporting operations, or export sales percentage, either an indirect or direct form could consider measuring firm quality (Myles Shaver, 2011; Love and Roper, 2015; Manova et al., 2015). Within the scenario of imperfections and information asymmetries in the capital market, it may reduce the negative effects of financial constraints on labor productivity. The rationale for this probable outcome is that productivity is increased as a consequence of up-rising exporting operations since international markets have tougher competition that may give momentum to firms to develop both processes and products to achieve competitive advantage (Ganotakis and Love, 2012).

Furthermore, access to finance plays a crucial role in achieving business objectives such as firm performance and sustainable growth (Shariff et al., 2017; Chima et al., 2021). González Martinez (2014) along with De Vries and Duque (2018) agreed with (Shariff et al., 2017) and showed that SMEs’ success and sustainable growth were mostly driven by easy access to finance. Han and Gu (2021) concluded financial inclusion; external finance impacts are significant and positively associated with innovation performance of Chinese high-tech firms. Again, Canton et al. (2013) and Hewa Wellalage et al. (2020) mentioned that national macroeconomic conditions, national financial sector development and regulation, and the quality of national institutions influence both enterprises’ access to credit and firm performance. Ferrando and Ruggieri (2018) argued that in Euro zones, access to external finance is not only a constraint but also negatively associates with the firms’ financial structure and the overall productivity factor. Although according to the credit disbursements for SMEs in Bangladesh are noticeably improved, more than 70% of SMEs still have no access to formal funding (Alauddin and Chowdhury, 2015). Among the companies polled, 68.6% of small- and 44.7% of medium-sized companies are restricted by financial resources (Hubbard, 2012). A study undertaken by IFC (2013) indicates the credit gap in current outstanding SME loans is 1.8 trillion USD (equivalent to 2 trillion USD).

There is still a large credit gap and unmet demand for formal credit in Bangladesh, even for those who have access to it. So, therefore, access to external finance for most of the Bangladeshi SMEs is still a far cry. A few research conducted to investigate the relationship between access to finance and SMEs in Bangladesh. Aziz and Siddique (2016) revealed in their research that access to external finance represents a central pillar for SMEs’ growth in Bangladesh. Hossain et al. (2018) discussed some solutions to mitigate credit risk–credit supply gap to develop SME performance in Bangladesh. However, the association between financial access and firms’ quality and how their interaction affects firms’ performance *via* labor productivity are still untapped. Moreover, these above-mentioned studies did not apply novel econometric method often consider for designing pragmatic policy recommendations. Therefore, this article tends to overcome this gap.

From these above-mentioned viewpoints, this article aims to establish the causal effect of both access to finance classified as financial accessed through bank loan acceptance and financial obstacle through owner’s perception, and firm quality denoted as export sales; on SME labor productivity, respectively, in Bangladesh. It is mandatory because SMEs encounter poor productivity issues as crucial challenges in lower- and middle-income countries. This article also studies the separate effects of financially accessed direct and indirect export-oriented SMEs on labor productivity.

The contributions of this article are multi-fold. Different from prior studies, this is the utmost and pioneering study that investigates the interaction between access to external finance and export sales, and labor productivity nexus in a cross-sectional framework in the context of Bangladesh. As SME can be credit accessed, having good investment projects is probably vital to enhance firm productivity *via* export sales and can alleviate the negative impact of credit constraints on labor productivity successively (Bellier et al., 2012). Second, it examines the individual impact of indirect and direct export sales on credit-accessed SMEs’ labor productivity.

Third, the incorporation of treatment effects *via* the propensity score matching (PSM) methodology makes a significant contribution to our approach. Sample data are used to develop comparable observations by SMEs and access to external finance to assume a treatment’s causal effects in an event and thus to alleviate the endogenous problem due to selection bias. The simultaneous interplay between access to external finance and firm productivity can reduce self-selection, as is the case in PSM. Finally, this present article extends extant literatures in a number of ways by examining interaction between access to external finance and export sales, and labor productivity in several aspects. The theoretical framework constructed captured core concepts of the association between access to finance and firm performance. Then, we investigate empirically the theoretical conjectures and determine the impact of access to finance on SME labor productivity, as well as the impact of exports through different channels.

The rest of the article is organized as follows. Section “Review of Related Literature and Hypothesis Development” presents a review of related literature and hypothesis development. Section “Data, Variables, and Empirical Model” lays out the data, variables, and empirical model. Section “Estimation Results” presents estimation results. Section “Discussion and Policy Implications” presents discussion and the policy implications. Section “Conclusion” concludes with limitations and avenues for future research.

## Review of Related Literature and Hypothesis Development

Small- and medium-sized enterprises have become the major factors in the Bangladesh economy by creating income-generating ability and improving competitiveness which has a direct impact on productivity (Carree and Thurik, 2010). However, obtaining loans from financial institutions to manage capital from external sources is a major constraint for most SMEs. Rather they depend more on owner’s equity as internal funds through retained earnings, the lack of access to external finance plays as constraints in such scenario (Romano et al., 2001; Abor and Biekpe, 2006). These SMEs need to manage external sources to invest in their operations to stay competitive and increase financial performance.

### Access to External Finance and Labor Productivity

Firm performance, denoted by labor productivity, is a crucial indicator of economic development since it is closely connected to economic growth and a fundamental factor of living standard, referring to the possibility of wealth creation (Turok and McGranahan, 2013; Harrison et al., 2014). Again, the respective ability to acquire external capital is also an essential element in the development of SMEs. There is a common understanding based on the prior literature that access to finance plays a vital role on labor productivity (Cheng et al., 2014; Bjuggren, 2018). Several authors in their literature conclude that access to finance is one of the key elements of labor growth. In a survey of 10,000 SMEs’ access to financing, in 80 different countries, González Martinez (2014) and De Vries and Duque (2018) concluded that SMEs’ success and sustainable growth were mostly driven by easy access to finance. In addition, 39% of small businesses mentioned funding as an important impediment. Similarly, a poll of the New York Federal Reserve Board found it difficult to secure finance for 49% of 670 SMEs (Rahman et al., 2017). However, an article conducted by Boermans and Willebrands (2018) explores the influence of financial limitations on business performance using a sample of SMEs, which are micro-financing institution customers (MFIs). This study helps in the literature by providing an alternative measure of financial constraints based on actual loans and borrowing behavior. To evaluate the link between financial limitations and productivity in employment, they use surveys of 615 entrepreneurs in Tanzania. The results reveal that financial constraints impede worker productivity and represent key obstacles to successful entrepreneurial activity, using OLS regression and PSM methodologies. In the same year, Ferrando and Ruggieri (2018) exhibit the relationship among firms’ financial structure, external finance access, and the overall productivity factor in several euro countries from 1995 to 2011. Using firm level data from the Bureau van Dijk-Amadeus database, they explored significantly negative estimation for total factor productivity (TFP) with financial constraint. The result recommends that removing hindrances to external finance access would probably stimulate firm productivity. The following hypothesis was generated for testing.

**Hypothesis 1:** Access to external finance is positively attached to SME labor productivity.

### Firm Quality and Labor Productivity

Malchow-Møller et al. (2015) distinguish services and manufacturing firms and explore the relationships between international trade and the long-run productivity growth using the extensive dataset of Danish companies from 2002 to 2008. Their results show that firms that started to export goods in this period had higher average growth in productivity than firms that did not export throughout this period. After the launch of export services, productivity growth also rises, though less significant and mainly for enterprises in the service industry. The article by Sharma and Mishra (2015) came out in the same year as Malchow-Møller et al. (2015) and looks at how a group of Indian manufacturing companies linked trade involvement to productivity performance throughout the period of 1994–2006. There are also statistical data to infer that the exporting and importing markets are more productive.

The study conducted by Taspinar (2010) found that export affects productivity for most of the countries at present. He compared Poland and Sweden export cases, applying causal multiple regression from 1990 to 2006. On the contrary, the author (Csordás, 2018) empirically investigated the interlink between exports and labor productivity. The results found that labor productivity has a significant negative impact due to reliance on metal and ores export. They summarized that a large percentage of ores and metals consumption can be considered as a hindrance to labor productivity. Fryges and Wagner (2021) conducted a study of 54 micro-econometrics, which indicate that German export-oriented manufacturing companies are more productive than non-exporting. But prior empirical researches indicate that exports may not inevitably enhance productivity. A similar negative outcome noted by Falk and Hagsten (2015) who examine the factors of Swedish small- and medium-sized companies’ export activity in the computer and business service industries. An interesting finding from their paper is that the intensity of the link between export probability and initial labor productivity is low when they control firm effects. The study therefore examines the following hypothesis.

**Hypothesis 2:** There is a significant impact of firm quality through export sales on labor productivity.

### Financial Access, Firm Quality, and Labor Productivity

The author found the linkage between external finance, export, and firm performance of 130,840 Chinese manufacturing companies, covering a time frame of 2001–2007 (Chen and Guariglia, 2013). The results recommend that both export characteristics and liquidity play a vital role in this linkage. However, firms having high liquidity showed a weak linkage between external finance and firm performance, particularly for both foreign and private firms. Altomonte et al. (2016) focus on the existing relationship among productivity, credit-rationed, and exports, adding a unique sample of seven European countries for the year of 2010. They showed that high productivity firms and exports are less likely to be financially constrained and better credit access positively interacts with productivity and more likelihood of exporting.

Similarly, Berman and Héricourt (2010) examine the role of financial determinants on the probability of both export percentage and exports of 5,000 production-oriented firms for 1998–2004 in 9 emerging and developing economies. This paper presents that liquidity access plays a vital role for firms in entering export markets. Contributing to the argument, Muûls (2012) investigated the role of financial constraints on existing exporters’ service destination and exporting decisions. The author used Belgium export data for the 7 years spanning period from 1999 to 2005, and derived results suggest that better financial access increases the likelihood of firms’ internalization as it aids to overcome fixed costs and sunk cost of entering a foreign market. Contrary to the above findings, Greenaway et al. (2007) applied a panel dataset of 9,299 United Kingdom production-oriented firms for 1993–2003, substantially controlling for unobservable and observable behavior to investigate the linkage between firms liquidity health for international market entry. This study implies that liquidity access has no significance on a firm’s decision to enter a foreign market. From these debates, we posit the following hypothesis.

**Hypothesis 3:** Firm quality through export sales has significant impact on labor productivity of credit-accessed firms.

### Credit-Access, Direct and Indirect Export, and Labor Productivity

Employing World Bank enterprise survey data operated in Turkey for the year of 2005, Abel-Koch (2013) exhibits that indirect exports are less profitable to minimize sunk cost of developing individual foreign distribution channel. This empirical analysis is robust to the acclimation of controlling other firm attributes and concludes that this failure has a predictable effect on the share of direct exports toward zero. Meanwhile, Crozet et al. (2013) also use French data in a different method. There, they calculate the total production factor to separate exports into quality (selling at a high price) and productivity-driven ones (which have a low price). Then, they examine the costs imposed by wholesalers in destination markets, which shows exports that are quality-based are cheaper if exported indirectly. Ahn et al. (2011) used Chinese business-level data and discover that enterprises choose endogenously between direct and indirect productivity-based exports and that only high productivity companies can pick the right to export directly with higher fixed costs. As a result, they also show that, according to distance and market size of destination countries, the share of indirect exports rises, with trade costs measured.

This study proposes a coherent theoretical framework that incorporates the two decisions – the export decision and the export channel decision (Fernández-Olmos and Díez-Vial, 2014). The purpose of this study is to investigate the relationship between exporting strategies, company efficiency, and downsizing. Using the resource-based perspective, they first investigate whether there is a link between export intensity and a firm’s proclivity to shrink. They used sample data of Spanish industrial enterprises between 1993 and 2016 period. The derived findings show that a firm’s level of involvement in overseas markets through exports has a negative effect on its inclination to downsize. Their findings also demonstrate that the propensity to downsize is lower in enterprises. On the other hand, the article by Lin (2017) investigates how financial limitations influence whether firms export directly or indirectly, and how this decision influences firm performance. The authors utilize the World Bank’s most recent Chinese private firm-level statistics, collected in 2011. The derived results indicate that indirect exporters are more exposed than direct exporters to financial limitations. Second, indirect exporters are not as productive as direct exporters. We propose the following hypothesis by summarizing the above-mentioned studies.

**Hypothesis 4:** Direct export sales shows higher level of labor productivity than indirect export for credit-accessed firms.

## Data, Variables, and Empirical Model

We used cross-sectional firm-level data for Bangladesh from the World Bank Enterprise survey for which there is access to external finance data, covering a total of 3,196 sample enterprises for the period 2007–2013. In extensive interviews with manufacturing and service-oriented enterprises, the survey data reflect business perceptions of the largest hurdle to firm performance and the relative significance of different restrictions on firm productivity. It is vital to note that this survey is limited to fully registered businesses with a minimum of five employees, initially in the manufacturing and service industries. The number of employees selected by WBES to distinguish small- and medium-sized firms is the most widely utilized criterion in Bangladesh (SME). Table 1 summarizes the definition of SMEs in Bangladesh (European Union Recommendation, 2003).

**TABLE 1:** Definition of SMEs.

Firm size	Number of employees
Micro-firms	<10
Small firms	<25
Medium firms	<250

*Source: Authors’ creation according to European Union Recommendation.*

### Data Analysis

Using the WBES sample dataset of SMEs, the study was carried out at both regional and national levels to explain potential differences. To lessen the impact of heteroscedasticity on the independent parameters, the interlink was stated by the ordinary least squares (OLS) regression equation along with robust standard error.


$$\begin{array}{c} L\,a\,b\,o\,r\,P\,r\,o\,d\,u\,c\,t\,i\,v\,i\,t\,y\\ =\beta_{1}+\beta_{2}\,\left(F\,i\,n\,a\,n\,c\,i\,a\,l\,A\,c\,c\,e\,s\,s\right)+\beta_{3}\,\left(F\,i\,n\,a\,n\,c\,i\,a\,l\,O\,b\,s\,t\,a\,c\,l\,e\right)\\ +\beta_{4}\,\left(F\,i\,r\,m\,Q\,u\,a\,l\,i\,t\,y\right)+\beta_{5}\,\left(F\,i\,r\,m\,C\,h\,a\,r\,a\,c\,t\,e\,r\,i\,s\,t\,i\,c\,s\right)\\ +\beta_{6}\,\left(O\,w\,n\,e\,r\,C\,h\,a\,r\,a\,c\,t\,e\,r\,i\,s\,t\,i\,c\,s\right)\\ +\beta_{7}\,\left(F\,i\,n\,a\,n\,c\,i\,a\,l\,A\,c\,c\,e\,s\,s*F\,i\,r\,m\,Q\,u\,a\,l\,i\,t\,y\right)\\ +\beta_{8}\,\left(F\,i\,n\,a\,n\,c\,i\,a\,l\,O\,b\,s\,t\,a\,c\,l\,e*F\,i\,r\,m\,Q\,u\,a\,l\,i\,t\,y\right)+\varepsilon \end{array}$$

The variables applied throughout the paper are then illustrated.

### Explained Variable: Labor Productivity

We employed labor productivity as a parameter of firm performance rather than TFP since TFP is frequently utilized as a residual and therefore prone to measurement error. This article measured labor productivity is mentioned below:


LaborProductivity=it[Employees-itEmployees](it-1)/[Employees+itEmployees](it-1)

### Explanatory Variable

Access to external finance is classified as financial access and financial obstacle as explanatory parameters to measure the outcome on firm performance through labor productivity. First, we apply dummy variables “Bank loan” to denote financially accessed SMEs due to invest in new and recent projects. The variables are individually coded as 0 if the firm rejected for bank loan. Similarly, it is individually denoted as one if the firm accepted for bank loan to overcome the shortage of finance in the last fiscal year. We further included the SME owner’s perception of “apply for bank loan” as an obstacle. This dummy variable of obstacle is encoded separately as 1 if they experience access to external finance through bank loan as minor or no obstacle to the present establishment activities and 0 otherwise if owners face moderate, major, or very severe obstacles to the firm’s operations (Love and Roper, 2015).

The firm’s quality is mentioned as the export sales of each firm as export signals more stable and reliable information about firm quality (Myles Shaver, 2011). As it is a more prominent and reliable unit across enterprises, export sales are chosen as alternative definitions of firm quality (Myles Shaver, 2011; Love and Roper, 2015). So, we apply direct and indirect export sales to account for the firm quality. We then apply dummy variables for direct export sales; encoded as 1 and 0 for the indirect export, and vice versa.

### Control Variables

The proposed econometric model used various control variables following preceding theoretical frameworks to obtain an unbiased estimator of the independent effect of the variables, particularly expressing the firm characteristics (Becchetti and Trovato, 2002; Nichter and Goldmark, 2009; Teal, 2016). The researchers have explained that new small- and medium-sized businesses are more volatile than enterprises and that they are more inclined to leave the marketplace. The age of firms can also have adverse repercussions on labor productivity (Coad et al., 2013; Cowling et al., 2018). Thus, by adjusting a permanent log converted variable, we regulate firm age. To ignore models concerning determination, the operating sector must be managed. This is why a dummy variable is used to illustrate whether companies are manufacturing or service (1 = service sector, 0 = other industries, for example, manufacture). Ownership once again affects long-term productivity, even if it will have a negative short-term impact (Kok et al., 2006). We, therefore, chose ownership to be a control variable, with the value of 1 for sole ownership and 0 for other forms of ownership. We also try to employ continuous and dichotomous variables to manage female ownership (Andriamahery and Qamruzzaman, 2022) and to evaluate the management experience of the owner (Aterido and Hallward-Driemeier, 2011).

### Propensity Score Matching for Treatment Effect

The effects of treatment allow the assessment of the causal effect on a sample output. This paper uses a matching technique to evaluate the treatment effects and construct a similar observation of SMEs’ access to external finance, as well as the likelihood of signal selection bias and endogeneity; yet, having similar observed qualities relative to constrained enterprises. The aim was to build pairs based on certain comparable samples, which are matched with untreated enterprises (bank loan accepted) and treated enterprises (bank loan rejected). This sort of matching is better than a random choice of comparable groups since the choice of small- and medium-sized enterprises with different features tends to minimize the bias (Abadie and Imbens, 2011; Rideout and Gray, 2013).

While matching techniques and regression depend on the freedom to impose causality, matching does not depend on the type of assertions of functional form that are commonly used in regressions. The matching also examines whether untreated observations have been made accessible for comparison in each treated sample. The recent economic survey shows that the reduction of selection bias can be achieved by preventing parameters and stating a common backup condition in the research according to sample data (Dixit et al., 2013; Karhunen and Huovari, 2015).

We adopted PSM strategy, suggesting that the likelihood of treatment confronts the features of samples (Chowdhury et al., 2021). The methodology is based on the likelihood of treatment derived from probit regression and is responsive to a set of observable characteristics (Berger et al., 2009). This technique has also been used to underwriting and loan bundling (Drucker and Puri, 2005). The propensity value is therefore considered an index function, which adds up to the broad range of observable attributes that influence the probability of the treatment (i.e., bank loan rejected). In other way, the PSM signals that the samples are subjected to the probability of a segment of the treatment group, which is:


P(X)=Pr(T= 1|X)

The authors suggested that under the estimation of conditional liberty (*Y_0_, Y_1_*)⊥*T*—*X*, the biases due to observable qualities can be avoided by conditioning independently on the propensity score, (*Y0, Y1*)⊥*T*—*P*(*X*) (Rosenbaum and Rubin, 1984; Benhabib et al., 2010). As researchers demonstrate, a common propensity would have extensive information regarding consistency choices, and optimal efficiency might be reached by PSM.

We implied an average treatment effect on the treated (ATET) of external finance access *via* no bank loan and severe obstacles to small and medium enterprises labor productivity. ATET is the difference between the average performance of the supported and unsupported companies when the unsupported company groups build matching units based on the propensity score level. When calculating the average accomplishment for those who have received treatment, the ATET criteria come into the consideration (Imbens and Wooldridge, 2009; Stojčić et al., 2019). Nevertheless, the probability of selecting two cells with the same propensity score value is not sufficient to evaluate the propensity score since the continuous variable features of the propensity score cannot be sufficient.

To counter this problem, several techniques have been proposed. These include PSM with a caliper specification and K-nearest neighbor matching (Caliendo and Kopeinig, 2008), the most widely used methods. Specifying the caliper enforcing a tolerance level on the maximum propensity score deviations can reduce bad matches, denoted as a caliper. We use a tolerance level of 0.05 to denote that we only want to assess an observation set if the absolute difference in PSM is less than 0.05 (Caliendo et al., 2008; Motta, 2020).

Although the discrepancy in estimations increases while undertaking a few matches, the benefit of a slight bias can be taken into account when specifying the caliper. We also emphasized unambiguously that residuals for independently and identically allocated data to be supplied as the robust default residuals for the projected ATET require achievable matching for treatment and control items (Abadie and Imbens, 2016; Hofert and Oldford, 2018).

Prior research has also shown that K-closest neighbor matching relates to K-nearest firms as a result of the propensity score. The K-option also imposes equilibrium between variance and bias, with a high value of K indicating low variance and high bias. We continue with the most popular option, 1 (Eliasson et al., 2012; Kobayashi, 2014; Murphy and Miller, 2019). However, in accordance with earlier work (Eliasson et al., 2012; Kobayashi, 2014), we also use 3 as the inception for K.

## Estimation Results

Table 2 presents the descriptive statistics accompanied by the mean and standard deviation of the independent, dependent, and control variables employed in estimation results of regression in this section. The table shows that labor productivity is positive with an average and standard deviation of 0.262 and 0.419, respectively. The mean financial access and financial obstacle are positive at 0.441 and 0.1183, accompanied by standard deviation of 0.497 and 1.177 subsequently. The close association between financial access and financial obstacles mean and standard deviation depicts the significance of labor productivity. Around 26% of firms are involved in exports with a standard deviation of 42.48. The average direct exports and indirect exports are positive of 20.28 and 5.468, along with a standard deviation of 38.92 and 21.18, respectively. The poor association between mean and standard deviation of direct and indirect exports depicts weak impact on dependent variable. In addition, the table exhibits that around 16% of service-oriented SMEs got accepted for bank loan. Proprietorship also shows an average of 0.510, accompanied by standard deviation of 0.500, where the maximum value is 1 and minimum 0.

**TABLE 2:** Summary of descriptive statistics.

VARIABLES	*N*	Mean	SD	Min	Max
Labor productivity	2,850	0.262	0.419	**−**0.805	1
Financial access	3,196	0.441	0.497	0	1
Financial obstacle	2,910	1.813	1.177	0	4
Exports	2,919	25.74	42.48	0	100
Direct exports	2,919	20.28	38.92	0	100
Indirect exports	2,919	5.468	21.18	0	100
Firm age	2,914	2.730	0.716	0	5.176
Service industry	3,196	0.155	0.362	0	1
Proprietorship	3,196	0.510	0.500	0	1
Managerial experience	2,907	16.88	10.02	0	60
Female ownership	2,922	0.182	0.386	0	1

*Source: Authors’ calculation.*

Table 3 presents the correlation matrix. As expected, there is the correlation coefficient of two indicators of access to external finance; financial access is positive and financial obstacle is negative, high (0.0789 and −0.0422) and statistically significant at 1 and 10% level, respectively. Export and labor productivity are strongly negatively correlated with a value of −0.147 at 1% significance level. The decomposition variable of exports, direct and indirect exports, which is credit-accessed exhibits negative but significantly correlated with labor productivity at 1% significance level, respectively, **−**0.111 and −0.0900.

**TABLE 3:** Correlation matrix.

	Labor productivity	Financial access	Financial obstacle	Exports	Direct exports	Indirect exports	Firm age	Service industry	Proprietorship	Managerial experience	Female ownership
Labor productivity	1										
Financial access	0.0789***	1									
Financial obstacle	−0.0422*	0.0732***	1								
Exports	−0.147***	−0.0566**	0.168***	1							
Direct exports	−0.111***	−0.0785***	0.136***	0.867***	1						
Indirect exports	−0.0900***	0.0302	0.0873***	0.415***	−0.0929***	1					
Firm age	−0.182***	–0.0103	−0.0417*	–0.0209	−0.0397*	0.0307	1				
Service industry	0.0632***	−0.125***	−0.161***	0.243**	−0.211***	−0.101***	–0.00110	1			
Proprietorship	0.0469*	0.108***	−0.172***	−0.337***	−0.297***	−0.130***	−0.0396*	0.121***	1		
Managerial experience	−0.205***	–0.00510	–0.0190	0.0329	0.00954	0.0484*	0.433***	–0.0174	−0.0603**	1	
Female ownership	−0.0646***	–0.0366	0.138***	0.242***	0.202***	0.115***	0.0581**	−0.0917***	−0.438***	0.0927***	1

*p < 0.05, **p < 0.01, ***p < 0.001.

*Source: Authors’ calculation.*

We proceed to operate the variance inflation factor (VIF) to confirm that multi-collinearity does not occur between explanatory and explained variables in Table 4. Table 4 exhibits that all VIF values are less than the cutoff values of 5, which denotes no significant multi-collinearity detected between dependent and independent variables. So, our result does not face a multi-collinearity issue.

**TABLE 4:** Detecting multi-collinearity using variance inflation factor among variables.

Variables	Labor productivity
Financial access	1.06
Financial obstacle	1.04
Direct export	1.20
Indirect export	1.07
Firm age	1.24
Service industry	1.13
Proprietorship	1.34
Managerial experience	1.24
Female ownership	1.26
Mean VIF	1.19

*Source: Authors’ calculation.*

Table 5 exhibits the regression model outcomes of the impact of access to external finance on labor productivity. The outcomes reported in columns 1 and 4 exhibit models with either one of the independent variables of interest (financial obstacle and financial access), and it shows both the financial obstacle and financial access are significantly positively related to labor growth. These outcomes are significant at the 1% significance level. This outcome is similar to the finding of Rupasingha and Wang (2017), which are bank loans and minor obstacles turned out to be significant in developing countries’ firm performance. Therefore, we recommend that policies toward investment in SME development and relaxation on SME may offset liquidity shortage from achieving investment facilities and eventually increase labor productivity.

**TABLE 5:** Interaction effect of export sales, access to finance, and labor productivity.

	(1)	(2)	(3)	(4)	(5)	(6)

VARIABLES	Labor productivity	Labor productivity	Labor productivity	Labor productivity	Labor productivity	Labor productivity
Financial access				0.0928*** (5.947)	0.0901*** (5.693	0.0938*** (5.025)
Financial obstacle	0.0563*** (3.577)	0.0608*** (3.893)	0.0736*** (4.030)			
Exports		−0.00144*** (−7.299)	−0.00123*** (−4.850)	−0.112** (−2.362)	−0.00151*** (−7.667)	−0.00144*** (−5.238)
Financial access × Exports						−0.000135 (−0.370)
Financial obstacle × Exports			−0.000493 (−1.351)			
Firm age	−0.0686*** (−5.604)	−0.0727*** (−5.982)	−0.0734*** (−6.039)	−0.0597*** (−4.993)	−0.0700*** (−5.785)	−0.0699*** (−5.779)
Service industry	0.0545*** (2.621)	0.0214 (1.010)	0.0197 (0.931)	0.0249 (1.180)	0.0477** (2.266)	0.0483** (2.288)
Proprietorship	0.0168 (0.964)	−0.0157 (−0.884)	−0.0160 (−0.902)	−0.0343* (−1.946)	−0.0125 (−0.709)	−0.0122 (−0.689)
Managerial experience	−0.00622*** (−7.312)	−0.00608*** (−7.205)	−0.00604*** (−7.143)	−0.00562*** (−6.689)	−0.00605*** (−7.140)	−0.00605*** (−7.138)
Female ownership	−0.0336 (−1.502)	−0.0171 (−0.769)	−0.0183 (−0.820)	−0.0131 (−0.598)	−0.0268 (−1.213)	−0.0267 (−1.212)
Constant	0.522*** (15.14)	0.586*** (16.64)	0.583*** (16.51)	0.411*** (11.66)	0.562*** (15.88)	0.561*** (15.66)
Observations	2,819	2,816	2,816	2,827	2,825	2,825
R-squared	0.062	0.079	0.080	0.109	0.084	0.085
*F* stat	30.74	34.57	30.48	31.36	37.14	32.50

*t-statistics in parentheses.*

***p < 0.01, **p < 0.05, *p < 0.1.

*Source: Authors’ calculation.*

Again, in columns 4 and 5, SMEs with higher firm quality, calculated by export sales percentage, have a significantly negative impact on labor productivity. However, the findings of the analysis indicate that SMEs with a higher level of export sales have lower labor productivity which is similar to the findings of Taspinar (2010). The probable reason for our different outcome could be that Bangladesh might not have sufficient knowledge and enough experience to utilize resources economically, so in the long run, the export becomes more costly than expected.

To justify the third investigation, columns 3 and 6 in Table 5 show the interaction effect between firm quality through export sales and access to finance. Our analysis shows that for a given export sales proportion, SMEs that have bank loans have lower labor productivity than firms having financial constraints due to the absence of bank loans. In another way, credit-accessed SMEs with lower entry to the international markets have negative labor productivity than constrained SMEs. However, there is no statistical significance found in these interaction results because export-oriented SMEs might have insufficient access to external finance. The reported results also support that age of firms and managerial experience are negatively significant at the 1% level. However, the only difference in service industry is positively significant at 1% and 5% levels. This highlights the significance of such control variables on labor productivity in the long run.

The results in Table 6 show the disintegration effect of export sales between direct and indirect exports. The results in columns 2 and 4 are explicit that both are statistically insignificant for direct and indirect exports. This analysis points out a negative association between direct and indirect exports with labor productivity for credit-accessed firms. In addition, there is a negative but statistically significant interaction between indirect export and access to capital as a perceived obstacle. This tells that the slope of indirect export sales proportion is smaller for SMEs that perceive financial access as a minor or no obstacle. However, the overall results for direct exports between financial access and obstacle are not statistically significant which is similar to the outcome of Rambocas et al. (2015). The probable reason behind that the exporters, with backing from indirect routes, are more likely to reach immediate financial goals; but the long-term goals are more effective by direct channels. We also found SMEs’ both firm age and managerial experiences are negatively significant to labor productivity. The service industry is also positively associated with labor productivity and in some cases shows a significant impact.

**TABLE 6:** Firm quality segmentation between direct and indirect exports.

	(1)	(2)	(3)	(4)

VARIABLES	Labor productivity	Labor productivity	Labor productivity	Labor productivity
Financial access			0.0906*** (5.722)	0.0937*** (5.024)
Financial obstacle	0.0602*** (3.852)	0.0740*** (4.057)		
Direct exports	−0.00136*** (−6.324)	−0.00120*** (−4.267)	−0.00141*** (−6.597)	−0.00128*** (−4.284)
Indirect exports	−0.00175*** (−4.728)	−0.00130*** (−2.814)	−0.00190*** (−5.142)	−0.00211*** (−3.704)
Financial access × Direct exports				−0.000237 (−0.591)
Financial obstacle × Direct exports		−0.000356 (−0.892)		
Financial access × Indirect exports				0.000335 (0.454)
Financial obstacle × Indirect exports		−0.00124* (−1.651)		
Firm age	−0.0725*** (−5.976)	−0.0738*** (−6.074)	−0.0695*** (−5.746)	−0.0696*** (−5.751)
Service industry	0.0220 (1.040)	0.0204 (0.964)	0.0478** (2.270)	0.0485** (2.297)
Proprietorship	−0.0158 (−0.890)	−0.0161 (−0.912)	−0.0120 (−0.680)	−0.0116 (−0.658)
Managerial experience	−0.00607*** (−7.194)	−0.00599*** (−7.091)	−0.00602*** (−7.110)	−0.00601*** (−7.091)
Female ownership	−0.0171 (−0.773)	−0.0182 (−0.821)	−0.0263 (−1.193)	−0.0262 (−1.187)
Constant	0.585*** (16.64)	0.583*** (16.52)	0.560*** (15.80)	0.559*** (15.60)
Observations	2,820	2,820	2,825	2,825
R-squared	0.080	0.081	0.085	0.085
*F* stat	30.52	24.75	32.70	26.21

*t-statistics in parentheses.*

***p < 0.01, **p < 0.05, *p < 0.1.

*Source: Authors’ calculation.*

### Propensity Score Matching

Table 7 exhibits the average treatment effects for labor productivity to all SMEs without a bank loan and major financial obstacles where the coefficient of the financial access is approximately 0.102 and 0.029, respectively; the coefficient values are less than the average of if all SMEs had bank loans and had minor obstacles. No bank loan and for major obstacles, the coefficients are insignificant in all other matching results. If SMEs had been granted a bank loan and had enough access to external finance, the result would come significant for labor productivity to SMEs’ financial access. Financial obstacles related to obstacles intensity in external finance for SMEs’ labor productivity are positively insignificant in PSM and other matching results. These outcomes are statistically positively insignificant for both PSM methods employed that are matched with our findings. The article also depicts a regression on the matched sample in Table 7, which was described as the observations in the treated group (rejected bank loan and major obstacle) plus the observations in the control group that were matched to the treatment group after the matching.

**TABLE 7:** ATET estimates for access to external finance.

		PSM	PSM with caliper	1-Nearest neighbor	PSM 3-Nearest neighbor
Financial access	ATET	0.102 (5.055)	0.078234358 (4.70)	0.105084703 (4.74)	0.092730206 (4.96)
	N	2,832	2,832	2,832	2,832
Financial obstacle	ATET	0.029 (1.327)	0.061532926 (3.71)	0.057258631 (2.44)	0.056531097 (2.97)
	N	2,822	2,822	2,822	2,822

*Outcome variable: Labor productivity. T-statistics in parentheses in columns 1, 3, and 4. Standard errors for independent and identically distributed data in parentheses in column 2.*

*p < 0.10; **p < 0.05; ***p < 0.01.

*Source: Authors’ calculation.*

Table 8 presents the outcomes of regression in the matched sample. The disparity between both no bank loans is statistically negatively significant, and those with large or extreme financial barriers are negatively significant at a 1% significance level, respectively. Not surprisingly, a similar path is found in our reported results, assuring the previous matching findings. In general, the derived outcomes exhibit that the criteria of matching are effective as the results are the same between no bank loan and bank loan, and major and minor obstacles for the matched data samples, respectively. Since there is no great disparity throughout treatment levels, the average treatment effects and PSM findings are perceived as reliable based on this result.

**TABLE 8:** Probit regression of the matched sample for access to external finance.

	(1)	(2)	(3)	(4)
	Propensity matching	Propensity matching	Propensity matching	Propensity matching
VARIABLES	Labor productivity	Labor productivity	Labor productivity	Labor productivity
Financial access			−0.0681*** (−4.271)	−0.0916*** (−5.823)
Financial obstacle	−0.0633*** (−3.958)	−0.0553*** (−3.515)		
Direct exports		−0.00128*** (−5.942)		−0.00132*** (−6.150)
Indirect exports		−0.00164*** (−4.404)		−0.00178*** (−4.761)
Firm age		−0.0734*** (−6.058)		−0.0701*** (−5.798)
Service industry		0.0449** (2.542)		0.0667*** (3.840)
Proprietorship		−0.0164 (−0.928)		−0.0120 (−0.679)
Managerial experience		−0.00606*** (−7.198)		−0.00602*** (−7.183)
Female ownership		−0.0157 (−0.708)		−0.0245 (−1.110)
Constant	0.301*** (24.52)	0.633*** (17.78)	0.303*** (24.90)	0.639*** (18.18)
Observations	2,822	2,822	2,828	2,828
R-squared	0.006	0.082	0.006	0.089
*F* stat	15.66	31.35	18.24	34.25

*t-statistics in parentheses.*

***p < 0.01, **p < 0.05, *p < 0.1.

*Source: Authors’ calculation.*

## Discussion and Policy Implications

This article is one of the first initiatives to investigate the interaction between access to external finance and export sales, and labor productivity nexus in a cross-sectional framework in the context of Bangladesh. Our outcome also depicts the individual impact of indirect and direct export sales on credit-accessed SMEs’ labor productivity. In addition, we have also examined the individual effect of both external finance access and firms’ export sales, on labor productivity. At first, this result exhibits that external finance access is a potential explanation for higher SME labor productivity in developing countries such as Bangladesh. This outcome is in line with the study by González Martinez (2014) and De Vries and Duque (2018) and reinforces that access to external finance is a significantly positive function of labor productivity growth in the SME industry.

Again, our results confirm that financially accessed SMEs along with a higher level of export sales have lower labor productivity. In 2021, this article also concluded a similar outcome for export effects on German firms’ labor productivity (Fryges and Wagner, 2021). Our result regarding firm quality and lower labor productivity is also consistent with the study by Falk and Hagsten (2015) in Sweden. This paper indicates that other external determinants obstruct the positive effect of export on firm labor productivity. These include high sunk costs incurred to enter export markets, inefficient allocation of working capital, absorbs excessive resources, lack of knowledge on target markets, lack of product innovations, and internationally uncompetitive capacity. The negative impact in our analysis indicates that SMEs with higher marginal cost are less productive enough to cover the sunk cost, such as R&D and promotional expenses, negotiating with potential foreign partners, customizing product attributes, among others. It also indicates that service-oriented firms rely more on research and development rather than resources and low labor available in local economy. Firms can improve their productivity through “learning by exporting” that exposition to external knowledge flow and technical efficiency (Eliasson et al., 2012).

Furthermore, our outcomes confirm that interaction effect between access to external finance and export sales is insignificant to SME labor productivity. Although probable reasons for this insignificant outcome were not examined in this article, possible explanations can be outlined from the prior article’s contributions. In 2013, the study by Chen and Guariglia (2013) also concluded a similar result of insignificant labor productivity in Chinese manufacturing firms. Chen and Guariglia (2013) argued that inefficient distribution of access to external finance channels on export-oriented SMEs causes insignificant labor productivity. The author recommended that a financial system should be developed, particularly bonds and stock markets to ensure more finance channels toward firms’ productivity. The result depicts in this article is also in line with the study by Greenaway et al. (2007) on the interaction between access to external finance and export sales in the United Kingdom market.

Our final findings relate to the individual impact of direct and indirect export sales on credit-accessed SMEs’ labor productivity. Direct exports’ impact on credit-accessed SMEs’ labor productivity is insignificant in our article. However, our outcome demonstrates that even minor obstacles on financially accessed indirect exports firms could indicate lower labor productivity in Bangladesh. Lejpras (2019) explained that service-oriented SMEs are less likely to engage in direct export, on average, lower labor productivity. In the same year, Chan (2019) argued that firms with insufficient liquidity prefer to adopt indirect exporting through an intermediate channel to lower fixed or upfront expenses. Due to the insignificant effect of direct export on firms’ productivity, our final outcomes tentatively recommend that external knowledge sourcing is crucial to develop innovativeness and internal knowledge for service-oriented SMEs.

Several policies can be drawn from the results of the current article to alleviate the negative impact of financially accessed firms’ exports on labor productivity in Bangladesh. It recommends that less productive firms should engage in exports through indirect channels, and firm managers should concentrate on external knowledge sourcing to improve marketing and product innovations to facilitate export channels to achieve international competitiveness. Existing policies of the Bangladeshi financial market should be assessed carefully and encourage stock and bond markets to participate in ensuring more sustainable external finance channels for enhancing SME labor productivity and sustainable economic growth. Furthermore, the government should induce new programs such as tax rebates on export items, flat exchange rates, and subsidized interest rates for bank loans to promote export through sustainable credit-accessed SMEs in Bangladesh. The business environment in Bangladesh changed in 2021, when businesses were in an early recovery phase following the ease of COVID-19 contamination. The current report showed that limited access to financing is one of the most problematic factors which indicate that firm performance suffered a lot due to this factor. The policies recommended from above findings will be beneficial to impede this problematic business factor and to ensure sustainable firm performance.

## Conclusion

The goal of this article was to establish the causal effect of both financial access and firm quality on SME labor productivity, respectively, in Bangladesh. This study also investigates the individual impact of financially accessed direct and indirect export-oriented SMEs on labor productivity, employing WBES cross-sectional firm-level data of 3,196 Bangladeshi firms for 2007–2013. OLS model and the PSM method were applied to examine the causal effect of the selected variables on SME labor productivity. The findings of our empirical results can be summarized as follows. The results depict that access to external finance is positively significant to labor productivity. However, our finding explores a negative relationship between exports and SME labor productivity. This outcome indicates insufficient knowledge and lack of experience to utilize resources at the maximum level by SMEs. The findings also exhibit no statistical significance in the interaction effect between firm quality and access to finance results on labor productivity because export-oriented SMEs might have insufficient access to external finance.

Furthermore, the study anticipates novel empirical support that the disintegration effect of export sales between direct and indirect exports with labor productivity for credit-accessed firms is also found statistically insignificant in our analysis. The article summary provides a useful supplement for extant literature on access to external finance and firm quality of SMEs’ inter-relation effects on firms’ performance in the context of lower- and middle-income countries.

However, the inclusion of lack of acclimation factors while comparing the linkage between external financing, export sales, and labor productivity must be acknowledged as a shortcoming of this work. Future research avenues can be expanded by adding other external factors as control variables to justify whether these determinants have a significant effect. Another drawback of this research is that this paper only included labor productivity in Bangladesh, which may render the findings insignificant for other middle-income economies. Future research in this avenue can consider this issue by expanding the research methodology to include more emerging South-Asian countries. It would also be viable of exploring whether the interaction effect of export sales and access to external finance differs among South-Asian countries. Last but not the least, the unavailability of the updated WBES data is another shortcoming of this article. Although the addition of the most updated WBES data in this article is ample to support the findings, the updated version in WBES data can still be considered for upcoming research work.

## Data Availability Statement

The datasets presented in this study can be found in online repositories. The names of the repository/repositories and accession number(s) can be found below: https://data.worldbank.org/.

## Author Contributions

MASC and SC: conceptualization, investigation, and writing–original draft preparation. MASC, SC, and ZA: methodology. ZA: software and validation. MASC: formal analysis and resources. ZA and MASC: data curation. AA and MS: writing, reviewing, and editing. SC: visualization, supervision, and project administration. MS: funding acquisition. All authors have read and agreed to the published version of the manuscript.

## Conflict of Interest

The authors declare that the research was conducted in the absence of any commercial or financial relationships that could be construed as a potential conflict of interest.

## Publisher’s Note

All claims expressed in this article are solely those of the authors and do not necessarily represent those of their affiliated organizations, or those of the publisher, the editors and the reviewers. Any product that may be evaluated in this article, or claim that may be made by its manufacturer, is not guaranteed or endorsed by the publisher.
