# Supplementary material for: Assessing the Empirical Linkage Among Access to Finance, Firm Quality, and Firm Performance: New Insight From Bangladeshi SMEs’
Source: Front Psychol. 2022 Apr 27;13:865733. doi: 10.3389/fpsyg.2022.865733 (PMC9093049; doi:10.3389/fpsyg.2022.865733)
Supplement: Supplementary file 1 [file Table_1.DOCX]

Supplementary Material

**Table A1 Definition of Variables**

| Variable | Definition | Source |
| --- | --- | --- |
| **Dependent Variables** |  |  |
| Labor Productivity | Percentage growth of number of employees | WBES |
| **Independent Variables** |  |  |
| Financial Access | Dummy variable, equals to 1 if the firm receives new bank loan and 0 otherwise. | WBES |
| Financial Obstacle | Dummy variable, equals to 1 if the firm faces minor or no obstacle to the firm’s operations and 0 otherwise. | WBES |
| Direct Export | Dummy variable, equals to 1 if the firm involves in direct export and 0 otherwise. | WBES |
| Indirect Export | Dummy variable, equals to 1 if the firm involves in indirect export and 0 otherwise. | WBES |
| **Control Variables** |  |  |
| Firm Legal Status | Dummy variable, equals to 1 if firm is sole proprietorship and 0 otherwise. | WBES |
| Firm Sector Status | Dummy variable, equals to 1 if firm is service oriented and 0 otherwise. | WBES |
| Firm Ownership Status (Female) | Dummy variable, equals to 1 if firm owner is female and 0 otherwise. | WBES |
| Firm Age | Year of the survey - year of incorporation | WBES |
| Managerial Experience | Top manager’s working experience (year) | WBES |

Source: authors’ creation
